# Pennsylvania Hospital—Surgical Wards—Service of Dr. Norris

**Published:** 1850-12

**Authors:** 


*Pennsylvania Hospital—Surgical Wards—Service of* Dr. Norris.
*Cases discharged from Jan. 1st to Feb. 1st,* 1850.

| Disease. | Cured. | Average time under treatment in days | By request. | Average time in days under treatment. | Died. | Average time in days under treatment. | Tot. |
|---|---|---|---|---|---|---|---|
| Abscess - - | 0 | 0 | 1 | 60 | 0 | 0 | 1 |
| Amaurosis - | 0 | 0 | 1 | 86 | 0 | 0 | 1 |
| Burns - - | 2 | 49.5 | 0 | 0 | 0 | 0 | 2 |
| Contusion - | 1 | 19 | 0 | 0 | 0 | 0 | 1 |
| Fractures, 9 | | | | | | | |
| simple 6, viz.: | | | | | | | |
| Clavicle - | 1 | 23 | 0 | 0 | 0 | 0 | 1 |
| Humerus - | 1 | 53 | 0 | 0 | 0 | 0 | 1 |
| Condyle of humerus | 1 | 38 | 0 | 0 | 0 | 0 | 1 |
| Radius - | 2 | 45.5 | 1 | 10 | 0 | 0 | 3 |
| Leg - - | 1 | 89 | 0 | 0 | 0 | 0 | 1 |
| Compound, 3, viz.: | | | | | | | |
| Facial bones - | 1 | 28 | 0 | 0 | 0 | 0 | 1 |
| Skull - - | 1 | 124 | 0 | 0 | 0 | 0 | 1 |
| Leg - - | 1 | 79 | 0 | 0 | 0 | 0 | 1 |
| Furunculus - | 0 | 0 | 1 | 2 | 0 | 0 | 1 |
| Gonorrhœa - | 1 | 14 | 0 | 0 | 0 | 0 | 1 |
| Hæmorrhois - | 1 | 8 | 0 | 0 | 0 | 0 | 1 |
| Hydrocele - | 0 | 0 | 1 | 1 | 0 | 0 | 1 |
| Inflammat'n of leg | 1 | 20 | 0 | 0 | 0 | 0 | 1 |
| " ankle | 1 | 41 | 0 | 0 | 0 | 0 | 1 |
| Necrosis - - | 0 | 0 | 1 | 48 | 0 | 0 | 1 |
| Onychia - - | 1 | 19 | 0 | 0 | 0 | 0 | 1 |
| Ophthalmia - | 2* | 31.5 | 0 | 0 | 0 | 0 | 2 |
| Ruptured vein - | 1 | 25 | 0 | 0 | 0 | 0 | 1 |
| Sub-luxation - | 1 | 54 | 0 | 0 | 0 | 0 | 1 |
| Syphilis - - | 6 | 35.3 | 1 | 61 | 1 | 82 | 8 |
| Tumor - - | 1 | 20 | 0 | 0 | 0 | 0 | 1 |
| Ulcer - - | 2 | 24.5 | 0 | 0 | 0 | 0 | 2 |
| Wounds 6, viz.: | | | | | | | |
| Incised - | 3† | 17 | 0 | 0 | 0 | 0 | 3 |
| Punctured - | 2† | 18 | 0 | 0 | | 1 | 3 |
| | 35 | | 7 | | 2 | | 44 |

* In one of these an abscess of the cornea also existed.
† One of these of the abdomen.

